# Comparison of Laser-Sintered and Milled Patient-Specific Reconstruction Plates for Complications and Outcomes in Mandibular Defects—Comparative Analysis of a Single-Center Cohort

**DOI:** 10.3390/jpm13040648

**Published:** 2023-04-09

**Authors:** Alexander Hagmann, Robert Schappacher, Sebastian Pietzka, Andreas Sakkas, Mario Scheurer, Alexander Schramm, Frank Wilde, Marcel Ebeling

**Affiliations:** 1 Medical School, University Hospital Ulm, Albert-Einstein-Allee 23, 89081 Ulm, Germany; 2 Department of Oral and Plastic Maxillofacial Surgery, Marienhospital, Böheimstraße 37, 70199 Stuttgart, Germany; 3 Department of Oral and Plastic Maxillofacial Surgery, Military Hospital Ulm, Academic Hospital of the University of Ulm, Oberer Eselsberg 40, 89081 Ulm, Germany; 4 Department of Oral and Maxillofacial Surgery, University Hospital Ulm, Albert-Einstein-Allee 10, 89081 Ulm, Germany

**Keywords:** laser-sintered patient-specific reconstruction plates, milled patient-specific reconstruction plates, CAD/CAM, mandibular defects

## Abstract

CAD/CAM-manufactured implants are increasingly becoming the standard in current therapy. The question of whether the manufacturing-related rougher surface of selective laser fusion plates compared to milled, smoother reconstruction plates leads to increased postoperative complications such as infections, plate exposure, and fistulas has not yet been determined. A retrospective analysis of 98 patients who underwent surgical treatment with either a selective laser fusion plate or a milled reconstruction plate at our hospital was performed. The only significant predictors of the revision risk were the operation time and use of antiresorptive medication. In the KLS Martin^®^ group, the risk of revision decreased by approximately 20% for each additional hour by which the operation time was increased (OR = 0.81). In the Depuy Synthes^®^ group, the risk of revision increased by approximately 11% with each additional hour of operative time (OR = 0.81 × 1.37 = 1.11). Both groups showed no significant differences in the number of necessary revision surgeries as well as inpatient complications. In summary, we can say that the assumption that additively manufactured reconstruction plates have a rougher surface due to selective laser melting and thus make plaque accumulation and revisions more likely has not been confirmed. Overall, it seems imperative to select further studies regarding the clinical outcome depending on the selected plate system.

## 1. Introduction

Alloplastic reconstructions of the mandible after mandibular resections due to tumor diseases, osteoradionecrosis, or drug-associated jaw necrosis remain one of the most challenging tasks of craniomaxillofacial surgery [1,2]. Difficulties are caused on the one hand by the anatomical variability of the region to be replaced, and on the other hand by the complex mandibular movements, which are significantly responsible for basic functions such as chewing, swallowing, phonation, as well as facial expressions [2]. The development of CAD-CAM techniques (computer-aided design/computer-aided manufacturing) offers the possibility of achieving true-to-the-original contours of the resected bone based on preoperative CT data sets [3]. A distinction is made between conventional hand-bent mandibular reconstruction plates, which are pre-bent preoperatively on the basis of stereolithographic models, and patient-specific mandibular reconstruction plates, which can be individually manufactured by subtractive or additive manufacturing methods without the need to bend the plate [1]. It is known that the mechanical strength and stability of metals decrease with the degree and frequency of bending [4]. Various studies have shown that patient-specific mandibular reconstruction plates have a significant advantage over conventional hand-bent plates in terms of the risk of plate fracture [1,5]. In addition, the use of patient-specific implants increases the accuracy of mandibular reconstruction in terms of preserving the preoperative mandibular dimension, which leads to a reduction in the risk of postoperative neuromuscular dysfunction, such as cranio-mandibular dysfunction (CMD). Patient-specific mandibular reconstruction plates can be divided into two reconstruction systems according to the manufacturing process. A division can be made between additively and subtractively manufactured mandibular reconstruction plates. The in vitro study by Kasper et al. proved that the two reconstruction systems do not differ significantly in terms of elastic deformation behavior [5], but milling mandibular reconstruction plates is generally associated with design limitations [1]. Additive manufacturing techniques have been used to overcome these limitations [1]. A possible disadvantage of the additively manufactured mandibular reconstruction plates could be the rougher surface due to the manufacturing process, which, according to transferable observations, could lead to a quantitatively higher total amount of viable bacteria compared to smooth surfaces [6]. This assumption is supported by the in vitro study of Xie et al. which showed that porous Ti6Al4V discs produced by selective laser melting exhibited stronger bacterial adhesion than control groups with highly polished orthopedic Ti6Al4V discs [7]. Micro-roughness and micro-porosity on the surface of biomaterials provide niches that are too small to be accessible to large leukocytes but can be easily colonized by bacteria [8]. Moreover, if bacterial colonization occurs before tissue repair, the host’s defenses cannot prevent surface colonization and biofilm formation [8], which could cause infections to persist and, in the case of patient-specific mandibular reconstruction plates, necessitate their removal. To date, however, there have been few studies demonstrating the long-term maintenance of patient-specific mandibular reconstruction plates, let alone investigating any dependence of the risk of infection on differently manufactured plate systems. The question of whether additively manufactured mandibular reconstruction plates, due to their rougher surface, are more prone to postoperative complications that necessitate the removal of the patient-specific implant, is what we would like to investigate in the current retrospective study. We would also like to analyze whether the patient collective and the subgroups differ significantly in demographic and clinical characteristics, and thus describe any individual risk factors that may lead to the loss of the patient-specific implant.

## 2. Materials and Methods

The present retrospective monocentric study was conducted at the Department of Oral and Maxillofacial Surgery of the Military Hospital Ulm and approved by the responsible ethics committee of the University of Ulm (application no. 140/22). The department’s database was searched for patients who underwent mandibular reconstruction between July 2014 and May 2021 in accordance with the guidelines and the standard of care using patient-specific mandibular reconstruction plates. Pre- and postoperative patient records as well as intraoperative documents were searched and the searched parameters were collected in an Excel spreadsheet, which was designed according to the recommendations of the Institute for Epidemiology and Medical Biometry of the University of Ulm. The processing and evaluation of the data took place in secured networks. After the completion of the study, the data were completely randomized and anonymized. Inclusion criteria were patients with mandibular defects in whom mandibular reconstruction was planned and performed using patient-specific mandibular reconstruction plates from Depuy Synthes^®^ (Zuchwil, Switzerland) and KLS Martin^®^ (Tuttlingen, Germany). Depuy Synthes^®^ sells patient-specific mandibular reconstruction plates in collaboration with Materialise (Leuven, Belgium), which are CNC-milled (computerized numerical control) from a solid titanium piece after preoperative computer planning using CAD/CAM procedures [9]. Patient-specific mandibular reconstruction plates from KLS Martin^®^ are additively manufactured by selective laser melting of a Ti6Al4V powder and differ in this respect from the mandibular reconstruction plates from Depuy Synthes^®^ [9]. The reconstruction could be performed as purely alloplastic as well as by means of an additional cutaneous/osteocutaneous/osteomyocutaneous graft. Of the original *n* = 106 patients, *n* = 98 patients were included in the current retrospective study. In the eight excluded patients, mandibular reconstruction using patient-specific implants was planned but not performed. Reasons for this included an intraoperatively determined need for an alloplastic joint replacement, a performed decortication and subsequent sufficient stabilization using conventional reconstruction plates, a death, and patients’ negative attitudes towards surgical reconstruction. The patients who met the inclusion criteria were divided into cohorts according to the parameters of the plate system, inpatient complications, and revisions. The plate systems were divided into KLS Martin^®^ and Depuy Synthes^®^ according to the manufacturers of the patient-specific mandibular reconstruction plates. Regarding complications in the inpatient course, a distinction was made between complications and no-complications. Similarly, the patients were further divided into the cohorts of revisions and no-revisions.

### 2.1. Data Collection for the Study

In order to describe, compare, and evaluate the advantages and disadvantages of the different plate systems, clinical demographic characteristics were collected, which are presented in detail in Table 1, Table 2 and Table 3.

### 2.2. Statistical Analysis

To compare continuous variables (age, length of stay) among the groups, first the normality assumption was tested using the Shapiro–Wilk test, and the Levene’s test was applied for testing the equality of variances. If the normality assumptions were met for a given continuous variable, the *t*-test (with or without the equality of variances assumed) was applied to compare the distribution of this variable among the groups of patients. When the normality assumptions were not met, we used the asymptotic Brown–Mood median test to compare the continuous variables among the different groups of patients. Means and standard deviation were reported only for normally distributed data. Otherwise, medians and quartiles were reported instead.

To assess the risk factors for the binary response of complications and the revision incident, the logistic regression model was applied. For the length of stay, the gamma generalized linear regression model was used with the log link function.

For this purpose, a two-stage test procedure was first used to check whether there were significant differences regarding revisions within the groups/risk factors when the patients were separated according to the plate system, and the respective separation according to risk factors, i.e., the variables age, sex, ASA score, diabetes, smoking, alcohol, antiresorptive medication, immunomodulating medication, and length of operation. Subsequently, a separation into the subgroups KLS Martin^®^ and Depuy Synthes^®^ took place, and they in turn were examined to see whether one of the risk factors mentioned played a supporting role within the subgroup. In the case of the smoking risk factor, the first step was to determine whether smokers as a whole had a worse prognosis than non-smokers. The second step was to determine whether smokers who received the Depuy Synthes^®^ plate system had worse results in terms of the revision frequency in order to be able to make therapy recommendations or recommendations for the choice of plate systems that took into account the risk factors of individual patients. The procedure for the other risk factors was analogous to the smoking risk factor. The models were simplified using a backward stepwise procedure with the ***p***-value for removal ≥ 0.1. The initial and the final models were reported.

We interpreted only the results significant at the level of 0.05. In addition, we commented on the results significant at the level < 0.1 as some tendencies that needed to be further investigated, and as potentially underpowered.

All calculations were conducted using R studio ver. 2022.07.1.

## 3. Results

### 3.1. Baseline Demographic Features Comparison for System Groups

Of the total *n* = 98 patients, *n* = 54 (55.1%) received a mandibular reconstruction plate from Depuy Synthes^®^ and *n* = 44 (44.9%) received a mandibular reconstruction plate from KLS Martin^®^. A comparison of baseline demographic characteristics for the system groups showed that there were no significant differences between the groups regarding age or sex, so that the groups can be considered similar. It also showed that there was a significant difference in the number of patients within the system groups taking antiresorptive medication (*p* = 0.028). This number was increased in the Depuy Synthes^®^ cohort (Depuy Synthes^®^: 39%; KLS Martin^®^: 18%). Furthermore, there was also a significant difference (*p* = 0.006) in the use of immunomodulatory drugs, with a higher percentage in the Depuy Synthes^®^ cohort (Depuy Synthes^®^: 21%; KLS Martin^®^: 2%). There was an almost significant difference in the proportion of patients with diabetes mellitus (*p* = 0.053), which was increased in the KLS Martin^®^ cohort. There was also an almost significant difference in the distribution of patients regarding surgical indications (*p* = 0.059), as well as an almost significant difference in the distribution of length of stay between the groups (*p* = 0.053). Results that were not significant at the 0.05 level are cited as trends that potentially require further investigation, as mentioned earlier. Within the Depuy Synthes^®^ cohort, there were *n* = 20 (37%) revisions. Similarly, *n* = 16 (36%) revisions were necessary within the KLS Martin^®^ cohort. The comparison of both system groups thus showed no significant difference regarding revision frequency and revision indication (revision frequency: *p* = 1; revision indication: *p* = 0.839), although the Depuy Synthes^®^ cohort, as mentioned above, had significantly more patients with antiresorptive and immune-modelling drugs. The indication for plate revision was infection in *n* = 16 (29%) plates in the Depuy Synthes^®^ cohort. In KLS Martin^®^, the number of mandibular reconstruction plates removed due to infections was *n* = 11 (25%). Details and other parameters comparing the baseline demographics of both system groups can be found in Table 1.

### 3.2. Baseline Demographic Features Comparison for Complications/No-Complications Groups

Complications in the inpatient course affected a total of *n* = 38 (38.8%) patients. The comparison of the demographic baseline characteristics for the complications/no-complications cohorts revealed a significant difference in the distribution of patients regarding surgical indications (*p* = 0.001). There was also a significant difference in the percentage of patients taking antiresorptive medication (*p* = 0.007), which was greater in the no-complications cohort (complications: 16%; no-complications: 39%). Patients without inpatient complications therefore had significantly more antiresorptive medication. Looking at the proportion of revisions in both cohorts (complications: 32%; no-complications: 40%), there was no significant difference (*p* = 0.519). Details, as well as other parameters comparing the demographic baseline characteristics regarding inpatient complications, can be found in Table 2.

### 3.3. Baseline Demographic Features Comparison for Revisions/No-Revisions Groups

In accordance with the previous cohorts, the entire patient population was also divided into the cohort’s “revisions” and “no revisions”. According to the designation of the cohort, a revision that has taken place is the decisive parameter in the allocation of the patients. A total of *n* = 36 (36.7%) mandibular reconstruction plates had to be revised again. A comparison of the baseline demographic characteristics of both cohorts revealed a significant difference in the percentage of antiresorptive medication use (*p* = 0.002), which was increased in the no-revision cohort (revisions: 11%; no-revisions: 41%). In addition, there was also a significant difference in the distribution of patients regarding the indication for surgery (*p* = 0.013) between the revisions and the no-revisions cohorts. Accordingly, there were significantly more revisions for operations due to tumors and osteonecrosis. There was also a significant difference in the length of inpatient stay between the two cohorts (*p* = 0.026). Details, as well as other parameters in the comparison of the demographic baseline characteristics, can be found in Table 3.

### 3.4. Model for the Risk of Complication

Table 4A,B contain the original and final model for the risk of complications. Some interactions could not be included due to convergence problems. The variables with a *p* < 0.05 were the only significant predictors in Table 4A,B.

#### Risk Factors for Complications

The ASA score was not significant at the 0.05 level but was left in the model as it may have an indeterminate trend that should be investigated further.

**Figure 1:**
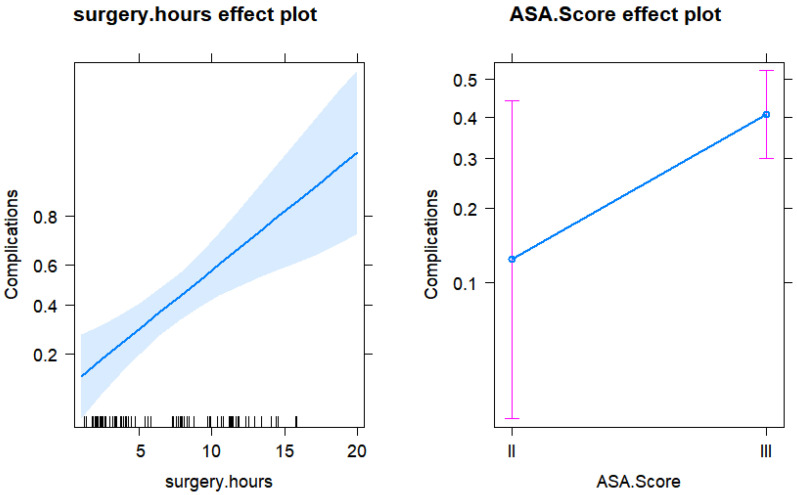
Effect plots for the surgery hours and ASA score from the final logistic regression model for the risk of complications.

**Figure 2:**
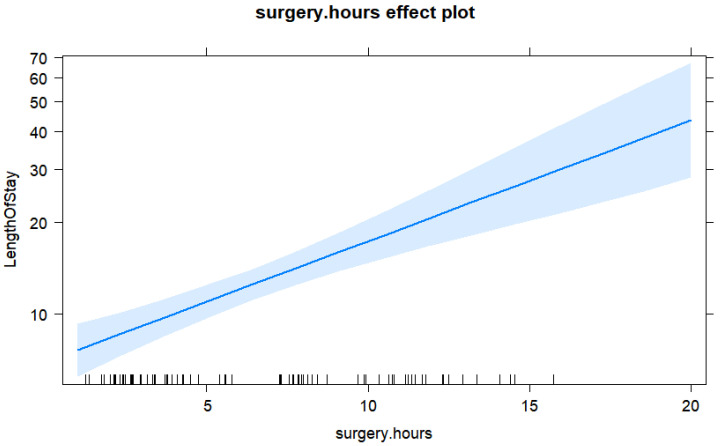
Effect plot for the length of surgery in hours prediction from the final Gamma regression model for the length of stay response.

The only significant predictor of the risk of complications in the final model was the number of hours of surgery. With each additional hour of surgery, the risk of complications increased by 27% (OR = 1.27) (Figure 1 and Figure 2).

### 3.5. Model for the Duration of the Inpatient Stay

Table 5A,B contain the original and final model for the length of stay. Some interactions could not be included due to convergence problems. The variables with a *p* < 0.05 were the only significant predictors in Table 5A,B.

#### Predictors for the Length of Stay

The only significant predictor of the length of stay in the final model was operation hours. Each additional hour of surgery increased the length of stay by 1 day.

### 3.6. Model for Risk of Revision

Table 6A,B contain the initial and final model for the chance of revisions. Some interactions could not be included due to convergence problems.

#### Risk Factors for the Revision

The variables with a *p* < 0.05 were the only significant predictors in Table 6A,B. The only significant predictors for the risk of revisions in the final model were surgery hours, antiresorptive medication, and group-surgery hours interaction term.

**Figure 3:**
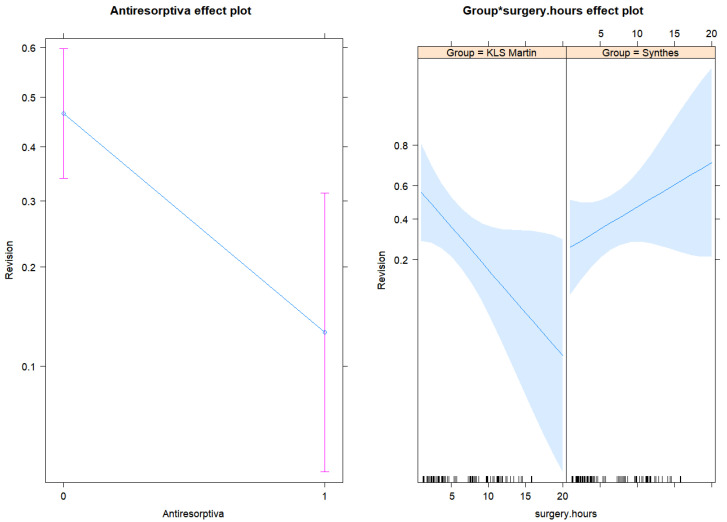
Effect plots for antiresorptive medication, the surgery hours, and group-surgery hours interaction term from the final logistic regression model for the risk of revisions.

As we can see from Figure 3, the effect of surgery time was the opposite in the two KLS^®^ and Depuy Synthes^®^ groups. In the KLS Martin^®^ group, with every additional one hour increase in the surgery time, the risk of revisions decreased by around 20% (OR = 0.81), having the antiresorptive medication group fixed. For the Depuy Synthes^®^ group, with every additional one hour increase in the surgery time, the risk of revisions increased by around 11% (OR = 0.81 × 1.37 = 1.11), having the antiresorptive medication group fixed. The antiresorptive medication group had a smaller risk of revision by 83% (OR = 0.17), as compared to the group without this medication, having the group variable and the time of surgery fixed (the same).

## 4. Discussion

The reasons for the removal of reconstruction plates in craniomaxillofacial surgery have already been described in detail in the literature [1,10,11]. In most cases, these include infections, osteomyelitis [10], exposed plates, pain [11], and fractures of the reconstruction plates [1]. It was also observed that in 90% of cases, the symptoms disappeared after removal of the plates [11]. In the present study, the proportion of mandibular reconstruction plates that had to be removed due to infections, abscesses, or fistulas was also the most frequent indication for plate revision. Thus, a total of 27.6% of all mandibular reconstruction plates were removed due to infections. Regarding the individual cohorts of Depuy Synthes^®^ and KLS Martin^®^, it can be concluded that the two plate systems do not differ significantly with regard to the number and indication of revisions (Table 1) and can be considered equivalent overall. However, the overall higher proportion of revisions due to infections with KLS Martin^®^ compared to the data in the literature is striking. In a retrospective analysis, Kreutzer et al. dealt with the prevalence and methods of the removal of patient-specific reconstruction plates in patients who had undergone mandibular reconstruction using KLS Martin^®^ microvascular-free fibula flaps and patient-specific titanium plates [12]. The proportion of additively manufactured KLS Martin^®^ mandibular reconstruction plates removed due to soft tissue complications (intraoral or extraoral plate exposure, wound healing disorders, fistulae) was only 12.2% in Kreutzer et al. [12], whereas the proportion in the current study was 25%, i.e., more than double. Even when adding the cases that had soft tissue complications (intraoral or extraoral plate exposure, wound healing disorders, fistulas) prior to the removal of the mandibular reconstruction plate, but had to be removed due to the fact that the mandibular reconstruction plate obstructed the insertion of implants or vestibuloplasty, the value of 20.4% by Kreutzer et al. was still below our revision rate [12]. However, it remains unclear why more revisions have occurred. The reasons for this are not apparent to the team of authors because possible explanations range from a majority of complicated cases to individual fates that ultimately remain inexplicable. In order to be able to answer this question to some extent, information on the degree of difficulty of the operation or the severity of the resection would have to be available, but this was not part of the parameters collected; therefore, this question remains unanswered. Of the included studies, only Kreutzer et al. contained data on revisions of KLS Martin^®^ mandibular reconstruction plates, so we are only able to compare the additively manufactured reconstruction plates. The percentage of fractured mandible reconstruction plates from KLS Martin^®^ determined in the present study was 4.5% and is comparable to the percentage of 3.1% reported in the literature [12]. Similarly, only 2% of the Depuy Synthes^®^ cohort had fractured plates. Both cohorts together resulted in 3.1% of the fractured plates in the current study. This low number, although not the main component of the present study, confirms the biomechanical advantages of patient-specific reconstruction plates. In support of this statement, studies with preoperatively pre-bent mandibular reconstruction plates showed a fracture rate of 16.6% [1], as well as in vitro studies of the biomechanical properties in which no fractures of patient-specific reconstruction plates occurred [5]. However, the present study also showed that fractures, albeit rare, can occur in patient-specific mandibular reconstruction plates and that reconstruction plates are subjected to more severe conditions in vivo than can be simulated in in vitro studies. Moreover, studies by Rana et al. [3] and Knitschke et al. [13] showed that postoperative complications are not uncommon even in operations with patient-specific mandibular reconstruction plates. Although these studies are dedicated to a different question, they do contain information and data on the frequency of postoperative complications such as wound-healing disorders and plate exposures after mandibular reconstructions using additively manufactured patient-specific implants. Within 6 months after surgery, Rana et al. found postoperative complications such as wound-healing disorders in 36.4% of cases, half of which also exposed the reconstruction plate [3]. In a comparison with conventional reconstruction plates, patient-specific reconstruction plates also performed worse with regard to plate exposures and plate-related fixation errors [13]. Plate expositions were found in 24.4% of the cases restored with a patient-specific mandibular reconstruction plate in Knitschke et al. while 15.6% had fixation errors [13]. Unfortunately, a direct comparison with the results of these retrospective studies is not possible, as it is not known whether the described or recorded complications resulted in the removal of the mandibular reconstruction plate, or whether existing complications could be treated by local antiseptic measures or antibiotics. Nevertheless, these data show that postoperative complications such as wound-healing disorders and plate exposure are also, or perhaps especially, found with patient-specific reconstruction plates, and their cause does not yet appear to be fully understood. However, it is certain that postoperative complications are often the cause of revisions, so we aimed to identify the predictors of revision risk. This showed that there were significantly more revisions in operations that were indicated due to tumors and osteoradionecrosis. This could be due, among other things, to the duration of the operation, which is linked to the respective indication, and which is longer in the case of tumor operations due to tumor resection and tumor-related neck dissection. In patients whose mandibular resection and reconstruction were due to osteoradionecrosis, the increased number of revisions is probably also due to the duration of the operation. Analogous to neck dissection, the postradiogenically scarred tissue requires more time for the preparation and visualization of anatomical structures. Although patients taking antiresorptive medication and requiring mandibular reconstruction due to AR/BPONJ may have had inflammatory tissues, this probably did not result in a large increase in operating time compared to neck dissections and postradiogenic scar tissue, so that the total operating time was shorter than is the case with operations due to tumors and osteoradionecrosis and could therefore be associated with fewer revisions. In addition, taking antiresorptives reduced the risk of revisions by 87%. The reasons for this are not apparent to the team of authors, so we suspect a coincidence behind this result. Another crucial finding of the current study suggests that the effect of surgery time is opposite in the two cohorts KLS Martin^®^ and Depuy Synthes^®^. Here, the risk of revision in the Depuy Synthes^®^ cohort increased by about 11% with each additional hour of surgery time, while the risk of revision in the KLS Martin^®^ cohort decreased by about 20% with each additional hour of surgery time. On the one hand, this could possibly have an influence on the choice of the plate system; on the other hand, this result could also have occurred purely by chance, as the team of authors found no explanation as to why the plate systems should behave so oppositely regarding the operation time. If this result did not occur by chance, the question of the operation time would have to be asked preoperatively to keep the revision risk as low as possible by choosing the appropriate plate system. However, this is only a continuation of the possible consequences of the result described above, which remains unexplained. The present study showed that, contrary to our expectations, additively manufactured mandibular reconstruction plates do not require significantly more revisions. Thus, the assumption that the rougher surface produced by selective laser melting is more easily colonized by bacteria seems to be wrong, or at least has no influence on the revision rate. Accordingly, additively manufactured mandibular reconstruction plates offer great potential for further innovations due to the flexible design, which is independent of blanks. However, it remains unclear why mandibular reconstruction plates have such high revision rates that are not related to fractures or material failure. There may be a correlation between plate thickness and the risk of soft tissue complication, such that the thickness of patient-specific reconstruction plates may favor plate expositions, as the soft tissue needs to be mobilized widely to cover the reconstruction plates and may be highly stretched. At the same time, however, there is the problem that a reduction in plate thickness will hardly be possible due to the biomechanical properties, as cases of plate fractures have already occurred in the current study, illustrating the mechanical stresses to which mandibular reconstruction plates are exposed. Another possibility, which, however, requires further investigation, is adjuvant irradiation, which, due to the large dimensions and thicknesses of the mandibular reconstruction plates, could have negative effects on the bone flaps due to scattered radiation occurring, which could lead to bone necrosis on the grafts with subsequent infections. This is why thinner and more delicate plate systems would be advantageous, especially for bone reconstructions. However, whether there is a connection and to what extent thinner and more delicate plate systems are suitable in mandibular reconstruction due to the biomechanical conditions remain questionable. Further investigations are necessary to clarify the exact background of the occurring infections and thus the necessary revisions. A multi-center approach may be appropriate to enlarge the patient population. Since both plate systems can basically be regarded as equivalent, there is also the possibility of a prospective study that considers the operation time when choosing the plate system and thus checks or confirms its influence on the revision rate. The limitations of the present study lie in the retrospective design, which was dictated by the size of the patient collective. Tendencies that were described as only almost significant in the results section require further investigation to determine the significance of these parameters.

Since this is a retrospective study and the length of the plates can no longer be reconstructed from the available records, we cannot provide any information on the plate length. However, since we basically have a standardized planning procedure for reconstruction plates, the plates should only differ marginally in length. Thus, since we cannot support this with data, we have excluded this part from the discussion.

In the future, technical innovations and further developments will also have to be considered. For example, KLS Martin^®^ has now succeeded in polishing the surface of its additively manufactured plates in such a way that no roughness or porosity is evident in the area of the plates’ surface and there is no longer any difference to the surface of a plate milled from a block, thanks to a special finishing process, the details of which are unfortunately not known to us and are not made public by the company. On the contrary, the new plates appear even shinier. To what extent such changes in the surface structure affect the complication rates cannot yet be estimated. However, based on our results, it could be assumed that no major improvements are to be expected, since our study showed that the surface of the plates does not seem to have a significant influence on the complication rate.

From the results of this study, it can be concluded that it makes no difference whether panels are manufactured additively or subtractively. Thus, factors other than the surface finish of the plates seem to be responsible for the postoperative complications. Therefore, further prospective multi-center studies are useful and necessary.

## 5. Conclusions

In summary, we can say that the assumption that additively manufactured reconstruction plates have a rougher surface due to selective laser melting and thus make plaque accumulation and revisions more likely has not been confirmed. The reasons why patient-specific mandibular reconstruction plates are prone to infection and plate exposure require further research, because only by identifying the cause can revisions be specifically avoided. If it is indeed due to the larger dimensions of patient-specific mandibular reconstruction plates, the development of a minimalist design or even the use of several individual patient-specific mini-plates may be appropriate, which should, however, be tested in advance for their suitability regarding biomechanical properties by means of in vitro studies. This is especially true since their use cannot represent an alternative to purely alloplastic mandibular reconstructions. However, other aspects, such as the already mentioned postoperative radiation, the altered soft tissue in osteoradionecrosis, and the incomplete debridement of necrotic bone must not be neglected in future examinations, as they could also be potential causes for occurring complications. Overall, it seems imperative to select further studies regarding the clinical outcome depending on the selected plate system.

## Figures and Tables

**Table 1:** Baseline demographic features comparison for system groups.

	Depuy Synthes^®^ (*n* = 54)	KLS Martin^®^ (*n* = 44)	*p*-Value
Age			
(Mean, SD)	59.48, 13.09	59.54, 10.20	0.597
Sex			
Males	30 (44%)	26 (59%)	
Females	24 (56%)	18 (41%)	0.838
ASA score			
II	7 (13%)	4 (9%)	
III	47 (87%)	40 (91%)	0.749
Diabetes (YES)	5 (7%)	11 (25%)	0.053
Smoking			
Current	25 (53%)	24 (67%)	
Ex	5 (10%)	0	
Never	17 (37%)	12 (33%)	0.211
Alcohol			
Daily	15 (34%)	15 (40%)	
Never	18 (41%)	17 (46%)	
Occasionally	11 (25%)	5 (14%)	0.475
Antiresorptive medication			
(YES)	21 (39%)	8 (18%)	0.028
Immunomodulating medication			
(YES)	11 (21%)	1 (2%)	0.006
Indication for the surgery			
1 = Tumor	20 (37%)	15 (34%)	
2 = Osteonecrosis	7 (13%)	11 (25%)	
3 = AR/BPONJ	21 (39%)	8 (18%)	
4 = Other indication	6 (11%)	10 (23%)	0.059
Complications			
(YES)	20 (37%)	18 (41%)	0.835
Revisions			
(YES)	20 (37%)	16 (36%)	1
Radiation			
(YES)	29 (54%)	22 (50%)	0.685
Chemo			
(YES)	24 (44%)	16 (36%)	0.534
Length of stay			
Median, Q1, Q3	9, 7, 18.5	13, 7, 19	0.053
Indication for Revision			
0 = No revision	34 (63%)	28 (64%)	
1 = Fistula/abscess/infection	16 (29%)	11 (25%)	
2 = Fracture	2 (2%)	2 (4.5%)	
3 = Relapse	2 (2%)	2 (4.5%)	
4 = Various	2 (4%)	1 (2%)	0.839

**Table 2:** Baseline demographic features comparison for complications/no-complications groups.

	Complications (*n* = 38)	No-Complications (*n* = 60)	*p*-Value
Age			
(Mean, SD)	65.6, 9.79	66.7, 9.94	0.594
Sex			
Males	24 (63%)	32 (53%)	
Females	14 (37%)	28 (47%)	0.404
ASA score			
II	2 (5%)	9 (15%)	
III	36 (95%)	51 (85%)	0.194
Diabetes (YES)	7 (18%)	9 (15%)	0.740
Smoking			
Current	19 (59%)	30 (59%)	
Ex	2 (6%)	3 (6%)	
Never	11 (34%)	18 (35%)	1
Alcohol			
Daily	14 (45%)	16 (32%)	
Never	11 (35%)	24 (48%)	
Occasionally	6 (20%)	10 (20%)	0.442
Antiresorptive medication			
(YES)	6 (16%)	23 (39%)	0.007
Immunomodulating medication			
(YES)	2 (5%)	10 (17%)	0.118
Indication for the surgery			
1 = Tumor	21 (55%)	14 (23%)	
2 = Osteonecrosis	9 (24%)	9 (15%)	
3 = AR/BPONJ	5 (13%)	14 (40%)	
4 = Other indication	3 (8%)	13 (22%)	0.001
Revisions			
(YES)	12 (32%)	24 (40%)	0.519
Radiation			
(YES)	18 (47%)	33 (55%)	0.535
Chemo			
(YES)	13 (34%)	27 (45%)	0.399
Length of stay			
Median, Q1, Q3	19, 13, 22	7, 6, 11.75	0.173
Indication for Revision			
0 = No revision	26 (68%)	26 (60%)	
1 = Fistula/abscess/infection	9 (24%)	18 (30%)	
2 = Fracture	0 (0%)	3 (5%)	
3 = Relapse	2 (5%)	1 (2%)	
4 = Various	1 (3%)	2 (3%)	0.544

**Table 3:** Baseline demographic features comparison for revisions/no-revisions groups.

	Revisions (*n* = 36)	No-Revisions (*n* = 62)	*p*-Value
Age			
(Mean, SD)	66.33, 9.38	66.24, 10.19	0.964
Sex			
Males	21 (42%)	35 (56%)	
Females	15 (58%)	27 (44%)	0.835
ASA score			
II	5 (14%)	6 (10%)	
III	31 (86%)	56 (90%)	0.745
Diabetes (YES)	6 (17%)	10 (16%)	1
Smoking			
Current	17 (65%)	32 (56%)	
Ex	1 (4%)	4 (7%)	
Never	8 (31%)	21 (37%)	0.740
Alcohol			
Daily	14 (50%)	16 (30%)	
Never	11 (39%)	24 (45%)	
Occasionally	3 (11%)	13 (25%)	0.167
Antiresorptive medication			
(YES)	4 (11%)	25 (41%)	0.002
Immunomodulating medication			
(YES)	4 (11%)	8 (13%)	1
Indication for the surgery			
1 = Tumor	17 (47%)	18 (29%)	
2 = Osteonecrosis	9 (25%)	9 (15%)	
3 = AR/BPONJ	25 (11%)	25 (40%)	
4 = Other indication	10 (17%)	10 (16%)	0.013
Complications			
(YES)	12 (33%)	26 (42%)	0.519
Radiation			
(YES)	17 (47%)	34 (55%)	0.532
Chemo			
(YES)	12 (33%)	28 (45%)	0.399
Length of stay			
Median, Q1, Q3	13, 9, 19	9, 6, 17.25	0.026

**Table 4:** Initial (**A**) and final (**B**) model for risk of complications.

**(A) Initial model for risk of complications**		
	**Complications**	
*Predictors*	*Odds Ratios*	*p*
(Intercept)	0.01 (0.00–22.83)	0.254
Group	24.78 (0.00–523425.07)	0.514
Surgery hours	1.55 (1.10–2.55)	0.033
Age	0.96 (0.87–1.07)	0.478
Gender [M]	1.35 (0.12–17.53)	0.805
ASA score [III]	8.61 (1.13–111.70)	0.055
Diabetes	0.59 (0.18–1.53)	0.308
Alcohol [never]	4.92 (0.40–82.59)	0.228
Alcohol [occasionally]	6.77 (0.24–309.39)	0.278
Smoking [ex]	2.10 (0.14–36.00)	0.586
Smoking [never]	4.42 (0.44–71.14)	0.232
Med immun	0.33 (0.04–2.13)	0.267
Antiresorptiva [1]	0.40 (0.01–8.88)	0.578
Group-surgery hours	0.73 (0.42–1.13)	0.196
Group-age	1.02 (0.90–1.17)	0.719
Group-gender [M]	0.49 (0.02–12.36)	0.666
Group-alcohol [never]	0.10 (0.00–2.85)	0.190
Group-alcohol [occasionally]	0.06 (0.00–5.01)	0.228
Group-smoking [never]	0.16 (0.01–2.89)	0.229
Group-Antiresorptives [1]	1.70 (0.05–102.76)	0.776
Observations	77	
R^2^ Tjur	0.306	
**(B) Final model for risk of complications**		
	**Complications**	
*Predictors*	*Odds Ratios*	*p*
(Intercept)	0.03 (0.00–0.19)	0.001
Surgery hours	1.27 (1.13–1.44)	<0.001
ASA score [III]	4.79 (0.96–38.23)	0.084
Observations	98	
R^2^ Tjur	0.190	

**Table 5:** Initial (**A**) and final (**B**) Gamma generalized regression model for the length of stay.

**(A) Initial model for the length of stay**		
	**Length of Stay**	
*Predictors*	*Estimates*	*p*
(Intercept)	22.49 (3.92–137.19)	<0.001
Group	0.20 (0.02–2.00)	0.159
Surgery hours	1.05 (0.98–1.13)	0.138
Age	0.98 (0.96–1.01)	0.201
Gender [M]	0.97 (0.59–1.56)	0.890
ASA score [III]	1.13 (0.68–1.82)	0.624
Diabetes	0.92 (0.75–1.15)	0.447
Alcohol [never]	1.01 (0.59–1.75)	0.967
Alcohol [occasionally]	1.25 (0.62–2.66)	0.537
Smoking [ex]	0.68 (0.39–1.25)	0.201
Smoking [never]	0.99 (0.62–1.62)	0.980
Med immun	0.74 (0.48–1.13)	0.148
Antiresorptiva [1]	0.71 (0.39–1.32)	0.263
Group-surgery hours	1.01 (0.92–1.10)	0.914
Group-age	1.03 (1.00–1.06)	0.040
Group-gender [M]	1.13 (0.57–2.26)	0.727
Group-alcohol [never]	0.67 (0.32–1.38)	0.292
Group-alcohol [occasionally]	0.74 (0.27–1.99)	0.550
Group-smoking [never]	0.71 (0.37–1.32)	0.265
Group-Antiresorptiva [1]	1.02 (0.47–2.18)	0.954
Observations	73	
R^2^ Nagelkerke	0.539	
**(B) Final model for the length of stay**		
	**Length of Stay**	
*Predictors*	*Estimates*	*p*
(Intercept)	6.93 (5.51–8.79)	<0.001
Surgery hours	1.10 (1.06–1.13)	<0.001
Observations	90	
R^2^ Nagelkerke	0.374	

**Table 6:** Initial (**A**) and final (**B**) model for risk of revision.

**(A) Initial model for risk of revision**		
	**Revision**	
*Predictors*	*Odds Ratios*	*p*
(Intercept)	62.58 (0.06–161236.37)	0.259
Group	0.00 (0.00–12.58)	0.161
Surgery hours	0.76 (0.55–1.00)	0.071
Age	0.96 (0.86–1.06)	0.419
Gender [M]	0.83 (0.10–6.35)	0.852
ASA score [III]	1.52 (0.21–13.08)	0.684
Diabetes	0.43 (0.13–1.15)	0.119
Alcohol [never]	0.74 (0.16–3.46)	0.699
Alcohol [occasionally]	0.58 (0.04–6.04)	0.664
Smoking [ex]	0.56 (0.02–11.48)	0.704
Smoking [never]	0.88 (0.11–6.17)	0.895
Med immun	1.20 (0.13–11.20)	0.868
Antiresorptiva [1]	0.12 (0.00–1.63)	0.140
Group-surgery hours	1.63 (1.11–2.52)	0.017
Group-age	1.05 (0.91–1.21)	0.511
Group-gender [M]	4.53 (0.27–94.21)	0.304
Group-smoking [never]	2.20 (0.14–40.70)	0.582
Group-Antiresorptiva [1]	1.12 (0.02–64.56)	0.952
Observations	77	
R^2^ Tjur	0.284	
**(B) Final model for risk of revision**		
	**Revision**	
*Predictors*	*Odds Ratios*	*p*
(Intercept)	2.69 (0.67–11.92)	0.171
Group	0.19 (0.03–1.20)	0.079
Surgery hours	0.81 (0.66–0.97)	0.035
Antiresorptiva [1]	0.17 (0.04–0.57)	0.007
Group-surgery hours	1.37 (1.08–1.77)	0.011
Observations	96	
R^2^ Tjur	0.180	

## Data Availability

Data is unavailable due to privacy restrictions.
